# Guillain-Barré Syndrome with Fatal Outcome during HIV-1-Seroconversion: A Case Report

**DOI:** 10.1155/2011/972096

**Published:** 2011-07-02

**Authors:** Emanuele Pontali, Marcello Feasi, Maria Paola Crisalli, Giovanni Cassola

**Affiliations:** Department of Infectious Diseases, Galliera Hospital, Via Volta, 16128 Genoa, Italy

## Abstract

Guillain-Barré syndrome (GBS) is an acute or subacute peripheral polyneuropathy characterized by symmetrical muscle weakness. Its occurrence has been reported during acute HIV seroconversion since 1985. Among HIV-infected subjects, GBS has generally a favourable outcome. We report a case of GBS with fatal outcome during HIV seroconversion.

## 1. Introduction

Guillain-Barré syndrome (GBS) is an acute or subacute peripheral polyneuropathy characterized by symmetrical muscle weakness occurring in the absence of identifiable causes of genetic, metabolic, or toxic origin. The characteristic presentation of the syndrome involves symmetrical paresis or paresthesia of an ascending nature, with reduction or loss of deep reflexes and variable autonomic dysfunction. Signs and symptoms observed in the course of GBS are extremely variable. The observed dysfunctions can be motor, sensory, or autonomic (Table 1) [1, 2]. Diagnostic approach to GBS is based on clinical, laboratory, and electrophysiological criteria (Table 1). Progressive motor weakness and areflexia are prime requirements for diagnosis. Cerebrospinal fluid analysis is the only laboratory criterion. Guillain-Barré syndrome onset is associated with a history of infection, mainly of viral origin. Many patients with GBS describe a febrile illness followed by an ascending paralysis occurring days to weeks later. Actually, symptoms are preceded by an antecedent event in about two thirds of cases. Respiratory infections are the commonest, reported in about 40% of cases within one month before the onset of the disease. About 20% of patients experience gastroenteritis as the antecedent cause [1]. Since 1985, several cases of the disease among HIV-infected patients have been reported, with GBS occurring concomitantly with HIV seroconversion or during the initial phases of infection [3–10]. In fact, HIV infection is one of the most common causes of this syndrome in African countries, India, and other developing countries. In addition, cases of GBS associated with the immunological reconstitution induced by the use of antiretroviral therapy have also been reported [11, 12]. GBS has frequently a favourable outcome in HIV-infected subjects [3–12].

To our knowledge, we report the first case of GBS with fatal outcome during HIV seroconversion.

## 2. Case Presentation

A 56-year-old man who had already been observed at our department for acute B (1988) and A (2000) hepatitis was evaluated at our Outpatient Unit (OPD). His medical history included viral encephalitis at age 6, chronic inflammatory bowel disease (treated with mesalazine), and past surgery for discal hernia. Homosexual intercourse with risk for HIV transmission was reported. He had tested HIV-negative about six-months before.

When presenting at our OPD, the patient reported the occurrence of fever (up to 38°C) and diffused maculo-papular rash about one month earlier. At that time, headache and intense pulsatory pain at shoulders had also been present for a few days. Blood tests showing evidence of probable acute viral infection had also been performed. During the three days before presentation at our OPD, he had been showing progressive asthenia, with increasing weakness at all limbs, dysarthria, and dysphagia. He was immediately hospitalised.

At entry he was confused, with amimic face, bilateral facial palsy (prevalent on the right), all limbs weakness (right side more than left), generalized hyporeflexia or areflexia at all limbs, anhysocoria (left > right), and heart rate 90 bpm, with blood pressure (BP) ascending in a couple of hours from 110/70 to 170/100. Skin rash was not anymore visible.

Among blood tests performed there was a mild increase in liver enzymes (complete results in Table 2). Antibodies for HIV-1 were detected, and confirmation test was positive. CD4+ lymphocyte count was 710/mm^3^ (45%), and viral load was 4,400 copies of HIV-RNA/mL.

One day after hospitalisation MRI scan of brain and truncus showed no specific signs (Table 2). The neurologist consultant then recommended lumbar puncture in the suspect of GBS. Lumbar puncture was performed on the third day. CSF examination showed 3 cells/mm^3^ and increased protein level (291 mg/dL), a pattern compatible with GBS [1]. Electroencephalogram was normal. BP was irregular, but gradually under control with antihypertensive drugs. Electrophysiological study was scheduled.

On the fifth day, the patient presented a worsening of limb weakness (both proximal and distal); anhysocoria was not anymore visible. He was started on intravenous immunoglobulins (IVIG) (400 mg/kg/day). Three days later he suddenly died because of cardiac arrest; prolonged resuscitation procedures were ineffective.

## 3. Discussion

The mechanisms proposed for GBS in HIV-1-infected patients include a direct action of HIV-1 on the nerves by neurotropic strains, or autoimmune mechanisms, with the formation of antibodies against myelin secondary to the abnormal immunoregulation determined by HIV infection [13]. The onset of GBS in this case coincided with the acute retroviral syndrome of HIV, during the phase of initial immune reconstitution following serologic conversion, as observed in other cases reported in the literature [6, 7]. Although GBS in HIV-infected subjects has generally a favourable outcome, rapid worsening of clinical conditions such as respiratory failure has been reported [7].

Both plasmapheresis and IVIG have been used to treat GBS in HIV-infected individuals [14–16]. The reported results have ranged from ineffective and mild benefit to good response [6, 14]. Nevertheless, IVIG currently remains the preferred choice in treating GBS. IVIG is preferred to plasmapheresis because of greater convenience, with overall comparable cost and equal efficacy [1, 17].

Twenty-five percent of all GBS patients require ventilator support of some type, and many of them require access to ICU [1]. Nevertheless, outcomes after ICU care were reported to be similar in HIV-positive and HIV-negative patients with GBS [18]. 

Unfortunately, in our case the rapid fatal evolution did not allow for ICU support. Cases with autonomic disturbances have been reported with similar evolution. Many deaths due to GBS are in fact attributed to autonomic disturbances; cardiac arrest being the most common cause, responsible for 20–30% of deaths [2]. Approximately 25% of deaths occur within the first week of onset of GBS.

Some conclusions can be drawn from this case. First, it is necessary to consider the possibility of GBS when patients are diagnosed with a symptomatic acute HIV syndrome. Patients could be advised to promptly report any neurological symptom or sign suggestive of GBS. This would allow for early diagnosis and proper timely management, including treatment and support. Second, the opposite is also true: in cases of GBS, HIV infection should be considered and searched. Last, but not less important, HIV-infected patients with GBS should be accurately monitored, because serious complications and fatal outcome of the disease are possible as in the HIV-uninfected.

## Figures and Tables

**Table 1:** Characteristics clinical features of Guillain-Barré syndrome and recommended diagnostic investigations.

Clinical features of Guillain-Barré syndrome		Recommended diagnostic investigations
Motor dysfunctions	Symmetrical limb weakness: proximal, distal or global; typically distal to proximal and rapidly ascending	Cerebrospinal fluid examination
	Muscle weakness (especially neck and respiratory muscles) or wasting (especially limbs)	Electrophysiological study
	Cranial nerve palsies: III–VII, IX–XII (most typical: facial palsy)	Stool culture for *C. jejuni *
	Areflexia	Serology to *C. jejuni*, Cytomegalovirus, Epstein-Barr Virus, Herpes Simplex Virus, HIV, *M. pneumoniae *
Sensory dysfunctions	Pain	Magnetic resonance imaging
	Numbness, paraesthesiae	Electrocardiogram
	Ataxia	Blood pressure monitoring
	Decrease or loss in proprioception, vibration, touch, and pain distally	Autonomic function tests
Autonomic dysfunctions	Sinus tachycardia and bradycardia	Antiganglioside antibodies
	Other cardiac arrhythmias	Biochemical screening: urea, electrolytes, liver enzymes
	Hypertension and postural hypotension	Full blood count
	Wide fluctuations of pulse and blood pressure	Erythrocyte sedimentation rate or C-reactive protein
	Hypersalivation	
	Anhydrosis or excessive sweating	
	Urinary sphincter disturbances	
	Gastric dysmotility	
	Constipation or diarrhea	
	Abnormal vasomotor tone causing venous pooling and facial flushing	
	Tonic pupils	
Others	Papilloedema	

**Table 2:** Results of diagnostic investigations during hospitalization.

Laboratory investigations		
Blood	alanine-aminotransferase (AST)	80 U/L (2 × ULN)
	aspartate-aminotransferase (ALT)	103 U/L (>2.5 ULN)
	Total bilirubin	1.8 mg/dL (normal < 1.2)
	lactic dehydrogenase (LDH)	603 U/L (normal < 460)
	HIV-Ab (including RIBA)	Positive
	CD4+ cell count and %	710/mL; 45%
	CD8+ cell count and %	595/mL; 37%
	CD4+/CD8+ ratio	1.2
	HIV viral load	4,400 copies of HIV-RNA/mL
	Hepatitis B virus markers	HBs-Ab, total HBcAb, and HBeAb: positive
	haemochrome, C-reactive protein, blood sugar, uraemia, creatinine, *γ*-glutamiltranspeptidase, alkaline phosphatase, creatine-phosphokinase, amylase, lipase, triglycerids, ions, protidogram, immunoglobulins (total IgG, IgM, IgA), Hepatitis C Virus-Ab, coagulation tests, complement fractions (C3 and C4), ANA, syphilis tests (TPHA and RPR), and urine analysis	Negative or within normal ranges
Cerebrospinal fluid	Cells	3/mL (all mononuclear cells)
	Proteins	291 mg/dL
	Protein analysis	Barrier damage with proportional increase in IgG and albumin
	HIV viral load	Not detectable
	Glucose, oligoclonal IgG bands, culture for bacteria and fungi, and syphilis tests (TPHA and RPR)	Negative or within normal ranges
